# Excessive Use of WeChat at Work Promotes Creativity: The Role of Knowledge Sharing, Psychological Strain

**DOI:** 10.3389/fpsyg.2021.571338

**Published:** 2021-03-18

**Authors:** Huiqin Zhang, Meng Wang, Meng Li, Xudong Chen

**Affiliations:** Business Administration, College of Management Science, Chengdu University of Technology, Chengdu, China

**Keywords:** excessive use, WeChat, knowledge sharing, psychological strain, creativity

## Abstract

The pervasive nature of social media can result in excessive use and addiction, but whether excessive use of social media is good or bad for individuals' creativity is unclear. This study explored the direct and indirect impact of excessive use of WeChat on individuals' creativity in workplace, focusing on how excessive use of WeChat promotes or restrains creativity through knowledge sharing and psychological strain. Based on the person–environment fit model and motivation theory, this study examined the three paths of excessive WeChat use affecting individuals' creativity in workplace. We used the structural equation model to test our research model. A survey of 364 employees revealed that excessive WeChat use directly promotes creativity and indirectly improves creativity via knowledge sharing, but excessive WeChat use does not lead to psychological strain. These findings, obtained by theoretically and empirically investigating the positive outcomes of excessive WeChat use, suggest an upside to excessive WeChat use. The implications and limitations of this study and future research on excessive-use behavior are also discussed.

## Introduction

Owing to the rapid development of mobile technology and popularity of smart devices, social media plays an important role in people's daily lives. Social media brings a great convenience as people can use it any time for entertainment, communication, and information. People can share ideas, pictures, and comments by interacting with others on social media (Alshahrani and Pennington, 2018). For example, individuals can upload their own edited content on social media, and others can comment on the shared content. Thus, social media provides great opportunities to communicate and share. Social media is used for personal activities as well as in the workplace. The China Internet Network Information Center (CNNIC) released the social media users' behavior research report, which found that enterprise/company employee respondents accounted for 31% of social media users, which was the highest percentage of all users [China Internet Network Information Center (CNNIC), 2016]. In organizations, employees use many social media platforms for sharing and communicating (Yu et al., 2018). Many studies have shown that using social media platforms can enhance individual job performance and productivity in the workplace (Aral et al., 2013; Moqbel et al., 2013; Alalwan et al., 2017; Cao and Ali, 2018). In addition, different forms of knowledge sharing on social media can help people develop ideas (Panahi et al., 2016a). However, frequently using social media may not always be beneficial. Some scholars have studied the impacts of excessive social media use. In the workplace, employees who excessively access social media may feel conflicted between work and life, as well as stress and reduced well-being (Zheng and Lee, 2016), which may negatively affect their job performance (Brooks, 2015). Yu et al. (2018) indicated that when social media use exceeds the optimal level at work, it will cause overload, which can affect job performance.

Recent studies played a lot attention to explore the use of social media. Bright et al. (2015) investigated Facebook users and found that privacy concerns and confidence are important predictors for social media fatigue. Sasaki et al. (2016) revealed that Japanese users changed their habits when they experienced information overload on Twitter. A few researchers have concentrated on Facebook use to explore core knowledge (Shiau et al., 2018) and privacy issues (Chou et al., 2019). Current studies mainly focused on the popular social media in Western countries such as Facebook and Twitter. Different from the most popular social media platforms in Western countries, Weibo, WeChat, QQ, and Qzone are the most popular social media platforms for communication and information seeking in China (Hussain et al., 2020). Despite the increase in social media research in recent years, few studies have focused on Weibo, WeChat, and QQ. WeChat is one of the most popular social media platforms in China and has more than 1 billion daily active users (Blog.Wechat, 2019). According to the WeChat behavior report in 2017, the login number for WeChat was 902 million users daily, and WeChat users sent 38 billion messages daily (Montag et al., 2018). These data reflect the increasing growth of WeChat. WeChat is popular, but less well-known worldwide. Because of cultural differences, research findings for Facebook and Twitter regarding the potential impacts of social media may not be applicable for WeChat.

WeChat is commonly used for widespread information sharing among employees because it is flexible and instantaneous. However, these advantages also have potential negative effects. Using social media enables effective information sharing, but processing a large amount of information can lead to information overload (Karr-Wisniewski and Lu, 2010), in which individuals lack sufficient capacity to process generated information (Eppler and Mengis, 2004). When using WeChat to communicate, most people belong to one or more chatting groups. Group chatting in WeChat helps people share knowledge simultaneously and talk to each other conveniently. Chatting groups generate large amounts of information, some of which requires individual replies. In the workplace, employees must allocate time and attention to process WeChat messages and complete work tasks. In other words, they must balance processing messages with completing their regular work. However, employees may face an imbalanced situation because of excessive access to WeChat. The person–environment fit model states that an imbalance between people and their surrounding environment can cause strain (Edwards and Van Harrison, 1993). Strain refers to the psychological outcomes of people's response to stress (Yu et al., 2018). When researching strain, many scholars focus on fatigue (Lee et al., 2016) and exhaustion (Maier et al., 2015b) when exploring social networking sites. Prior studies demonstrated that strain can negatively influence job performance (Cao and Yu, 2019). Emotional exhaustion is also negatively related to job performance (Choi et al., 2014). Furthermore, individual responses to a stressor can result in low creative performance (Rich, 2016). Thus, experiencing strain can result in bad organizational outcomes (Yu et al., 2018).

Whether social media use is good or bad for individuals is an interesting topic. Social media provides a platform for individuals to share information such as pictures, videos, emoticons, comments, and ideas. Individuals discussing, sharing, and combining information from various social interactions is beneficial for generating new ideas and knowledge (Marianna and Kalotina, 2015). However, spending a lot time and energy on social media to process messages leads to strain, which negatively affects creative performance. This poses an interesting question regarding whether excessive social media use facilitates creativity in the workplace. The multipurpose platform of WeChat enables message sending, social sharing, mobile payment, and city services in daily life. Over the past years, WeChat has developed rapidly with increasing active users and extended functions (Montag et al., 2018). WeChat as a personal social media platform has permeated the workplace. Individuals check the task assigned by superior or contact clients through WeChat, which unintentionally creates a stressful working environment for individuals because they have difficulty in separating personal activities and work tasks. Previous studies have explored excessive use of social media, but few have focused on the effect of excessive use of WeChat on individuals.

In light of this, we focus on excessive use of WeChat and its impact on individual creativity to determine whether excessive WeChat use promotes or restrains creativity and how it occurs. This study aims to answer the following research questions:

*RQ1*. What are the positive/negative consequences of excessive use of WeChat for users in workplace?
*RQ2*. How does the excessive use of WeChat influence individual creativity?

To answer the above research questions, this research contributed to social media research by focus on the specific social media application (WeChat) to examine the phenomenon of excessive use of WeChat in the workplace. We theoretically and empirically investigated the underlying mechanism of the different paths by which excessive use of WeChat influences individual creativity, extending previous research on excessive social media use. Drawing from the person–environment fit model and transactional theory of stress and coping, we develop and empirically test a theoretical model exploring the direct impact of excessive use of WeChat on individual creativity and how excessive use of WeChat indirectly influences individual creativity through knowledge sharing and psychological strain. We attempt to explore the positive consequences of excessive WeChat use through individual creativity, extending the understanding of the dark side of excessive social media use. This article is structured with seven sections; the following section provides the theoretical background and the background of excessive use of WeChat and creativity. The third section describes the research model and hypotheses, whereas the fourth section indicates the methodology that this study was conducted. The fifth section presents the results, followed by the last two sections, which report a discussion of the study and its conclusion.

## Theoretical Background and Related Research

### Personal–Environment Fit Model

The personal–environment (P-E) fit model assumes that people's capacity, preferences, and interests match the demands required by the environment, which is an equilibrium state (Edwards and Van Harrison, 1993). The P-E fit model is broadly defined as the adaptability of individuals to their surrounding environment and is widely used to explain issues in organizational research (Kristof-Brown et al., 2005). The P-E fit model is also a popular framework in stress research for understanding stress caused by the misfit between people's personal characteristics and their environment (Edwards and Cooper, 1990). If environmental patterns do not fit people's personal characteristics, people can become dissatisfied (Le et al., 2014). According to the P-E fit model, a break in the equilibrium state will generate stress and cause strain among individuals and their surrounding environment (Cooper et al., 2001). In short, the P-E fit model explains that the misfit between individuals' personal characteristics and the surrounding environment can result in strain (Edwards and Van Harrison, 1993). P-E misfits occur in two ways: either environmental supplies are inconsistent with individuals' personal characteristics, or individuals' personal characteristics do not match the demands of their environment (Edwards, 1996). In social media contexts, a misfit may exist between the user's energy and the demand of social media use (Lee et al., 2016). People's energy is limited, and heavy WeChat use in daily life can induce people to engage in excessive use, which may lead to strain. Therefore, this study used the P-E fit model to investigate the phenomenon of excessive WeChat use.

### Motivation Theory

Motivation theory emphasizes that motivation is the primary cause of individual's behavior, which explains how motivation influences individuals' behavior (Moon and Kim, 2001). In a previous study, two categories of individual motivations are extrinsic and intrinsic motivations. Extrinsic motivation factor includes some benefits obtained by an individual in the form of direct or indirect monetary compensation, such as bonus and promotion (Kwok and Gao, 2004). Intrinsic motivation is an inner drive that emphasizes the desire for achieving competence and self-determination, such as sense of belonging and reputation (Ryan and Deci, 2000). In the context of social network, information sharing, searching for friendship, obtaining social support, and entertainment are motivational forces for individuals utilizing social media to set up a social event with others online (Ridings and Gefen, 2004). The use of social media provides opportunity for repaid knowledge flow between people in different place (Panahi et al., 2016b) and creates a virtual space for facilitating knowledge-sharing activities (Kwahk and Park, 2016). In knowledge-sharing process, individuals can obtain some of the benefits such as reciprocity with knowledge providers (extrinsic motivation) and enjoyment in knowledge sharing (intrinsic motivation) (Hau et al., 2013). Individuals have certain knowledge reserve, with strong intention and enthusiasm to participate in knowledge management activities online. In recent years, scholars have attempted to use motivation theory to explain knowledge sharing in social media research. For example, based on motivation theory, Gilbert (2016) has explored Twitter as a learning tool to support learning in online communities and community of practice. The sense of reciprocity and altruism are extrinsic motivators, whereas personal satisfaction and reputation enhancing are intrinsic motivators for individuals to share knowledge online (Gilbert, 2016). Thus, this study utilized motivation theory to explain how excessive use of WeChat influence employees' knowledge-sharing behavior. Based on extrinsic and intrinsic motivation, individual contributes their energy and time on WeChat to provide or share knowledge and engage with others to access information.

### Excessive Use of WeChat

Many studies have focused on social media use for information sharing and communication in the workplace (Moskaliuk and Kimmerle, 2009; Ou and Davison, 2011; Van Zoonen et al., 2017). Schmidt et al. (2016) reported that employees' use of social media could gain information benefits through their connecting with work colleagues. Sheer and Rice (2017) indicated that employees rely heavily on mobile instant messaging to communicate with their work contacts. These studies demonstrate that social media is primarily used for work-related purposes in organizations. Social media was originally defined as a personal interaction platform, but its social affordance makes it difficult for individuals to engage only in personal activities whether in the workplace or elsewhere (Koch et al., 2012). For example, employees communicate with others on social media to exchange information for task-related purposes, as well as for socializing (Sun and Shang, 2014).

Social media connects individuals to family, friends, acquaintances, and colleagues anywhere at any time. To maintain the social relationships embedded in social networks and to gain support and belonging (Cao et al., 2016), individuals must frequently check their social media messages and respond as quickly as possible. Employees spend a lot time checking messages, which may distract them and influence their work progress. Prior research has shown that rational hedonic use of social media can increase employees' job satisfaction (Moqbel et al., 2013). However, because people are attracted by the nature of social media that emphasizes enjoyment, they often engage in intense use (Leftheriotis and Giannakos, 2014; Li et al., 2017). To satisfy their craving for pleasurable experiences, employees become tolerant to spending a lot of energy on social media, even in the workplace (Wang et al., 2015). Such use of social media has become a new domain that researchers are focusing on. Scholars describe this phenomenon as excessive use (Ndasauka et al., 2016), social media dependence (Wang et al., 2015), compulsive social media use (Aladwani and Almarzouq, 2016), and social network service addiction (Choi and Lim, 2016). Authors of previous studies believed that excessive use of social media is a symptom of an individual's problematic use or addiction (Deryakulu and Ursavaş, 2014). However, whether the potential effects of excessive social media use on individual job performance are good or bad remains unclear. Studies on the excessive use of specific social media platforms in the workplace are limited.

As WeChat is widely used, and its functions continue to expand, individuals tend to spend considerable time and energy using WeChat. For example, when individuals communicate with friends on WeChat to obtain suggestions, they might unconsciously check messages from other users sharing photographs or videos via the *moments* function. Individuals tend to participate more and intensely use WeChat. Researchers have termed this “WeChat use disorder” (Montag et al., 2018) or “WeChat addiction” (Li et al., 2018) and have explored the potential influences of this phenomenon. Li et al. (2018) demonstrated that WeChat addiction positively influences life satisfaction because people use it to strengthen friendships through sharing with each other. WeChat use is neither good nor bad; the positive or negative influence on individuals depends on what they use it for (Wen et al., 2016). WeChat addiction is related to how individuals use WeChat, as well as their personal characteristics. Hou et al. (2018) indicated that the personality trait of neuroticism is positively related to excessive WeChat use, whereas agreeableness negatively affects excessive WeChat use. These studies explored WeChat addiction or excessive use of WeChat on an individual level. However, studies on the related consequences of excessive and problematic use of WeChat in the workplace are scarce, and the true influence on employees' performance has not been systematically explored. Drawing on the definition of excessive social media use by Cao and Yu (2019), we define excessive use of WeChat as the degree to which individuals perceive that they spend too much time and energy on WeChat. This study explored the impact of excessive use of WeChat on employee creativity.

### Creativity

Creativity is increasingly considered a competitive advantage for organizations, and the knowledge that employees have and exchange is the basis of creativity (Rhee and Choi, 2017). Creativity is often defined as the generation of novel ideas (Marianna and Kalotina, 2015). In the workplace, most people accept the definition that explains creativity as new and improved ways of doing things, including process optimization and innovative products (Anderson et al., 2014). Workplace creativity includes the cognitive and behavioral processes of generating new ideas (Hughes et al., 2018). To maintain or enhance the effectiveness of responding to a changed environment, organizations must be creative at the individual, group, and organizational levels (Zhang et al., 2011). Most research on creativity has focused on individual-level factors such as employees' traits, abilities, and cognitive styles (Shalley et al., 2004) and organizational factors such as organizational culture (Ogbeibu et al., 2018) and leadership style (Hughes et al., 2018). Individual creativity performance as the basis of group and organizational creativity is influenced by factors other than individual personal characteristic factors. For example, social networks, which hide interindividual relationships (Marianna and Kalotina, 2015), affect people's creativity. Social media use has dramatically changed people's communication styles (Filo et al., 2015). Individuals can continuously and informally exchange their thoughts with others on social media. That means people can share and aggregate knowledge from various sources to create new meta-knowledge (Marianna and Kalotina, 2015), which helps create new ideas. Research has explored the relationship between social media use and individual creativity. For example, Korzynski et al. (2019) found that the use of social media facilitates employee creativity. Nevertheless, no studies have explored how social media use influences individual creativity in the context of excessive use. Therefore, this study explored the influence of social media use on individuals' creativity in the context of excessive use.

## Research Model and Hypotheses

### Excessive Use of WeChat and Strain

One of the core functions of WeChat is a messaging service that allows people to send text messages and share photographs, videos, and emoticons to individuals or groups. WeChat helps individuals connect with family, friends, acquaintances, colleagues, and others. As the number of messages from virtual friends in social media increases, individuals face the demand of processing many messages, frequent communication, and constant social requests (Yu et al., 2018). That means they must pay attention and reply to messages. Once the demand exceeds an individual's capability, it will lead to social media overload, including information, communication (Lee et al., 2016), and social overload (Maier et al., 2015a). This overload reflects the misfit between the surrounding environment and personal capability. From the P-E fit perspective, misfits between a person and the surrounding environment can result in strain (Edwards and Van Harrison, 1993). Thus, based on the above arguments, we propose the following hypothesis:

H1. Excessive use of WeChat significantly and positively affects strain.

### Strain and Creativity

The transactional theory of stress and coping states that stress processes cover stressors and strain; stressors are stimuli, whereas strain is individuals' psychological outcomes to stressors (Lazarus and Folkman, 1987). It explains the phenomenon of stress as a transaction, which require individual taking action (e.g., psychological reaction, behavioral responses) to cope with the imbalance between the environmental demands and individual capacity (Srivastava et al., 2015). In this study, the usage experience of excessive use on WeChat was the stressor. When individuals encounter misfit from demands of WeChat use that exceed their capability, they experience psychological strain (Hobfoll, 2001). Psychological strain refers to individuals' subjective feelings for stressors they face in their surrounding environment (Van Dyne et al., 2002). This requires spending time and energy to deal with stress. Thus, individuals may allocate fewer resources (such as time and energy) to other things rather than address stressors because their mental resources are limited. In this way, individuals are more likely to adopt common and simple ways of doing things while generating fewer creative ideas (Byron et al., 2010). Similarly, employees may lean upon habitual actions to complete work tasks and neglect more creative methods when they must exert more time and energy to cope with strain (Van Dyne et al., 2002). Therefore, we predict strain will lead to low creativity. Based on the above argument, we propose the following hypothesis:

H2. Strain significantly and negatively affects creativity.

### Excessive Use of WeChat and Knowledge Sharing

As a social media platform, WeChat enables users to release their own organized content, share photographs/videos through the *moments* function, and communicate with others. Others browse through the uploaded content and give their opinions by commenting below the content. Individuals can also put forward their own views of others' edited content (i.e., exchanging viewpoints). WeChat provides group chat for users to join different groups and interact with other group members. Most users belong to various chat groups, which are online communities in practice. They allow users to communicate with diverse group members and obtain knowledge simultaneously during communication (Tahir et al., 2019). Hence, individuals can manage the knowledge embedded in various interactions in virtual networks, which helps generate knowledge (Marianna and Kalotina, 2015). Related research emphasizes that individuals share experiences successfully with others only by spending time together communicating (Tahir et al., 2019). Experience is widely considered as tacit knowledge. Therefore, spending too much time and energy on WeChat may help people share and acquire knowledge. According to motivation theory, altruism indicated that individuals contribute their energy and time to provide or share knowledge with others, and some studies have proven that altruism is an important motivation to promote individuals' participation in knowledge sharing (Kwok and Gao, 2004). In short, excessive use of WeChat facilitates communication under the social function of WeChat, which enables exchanging and sharing knowledge. Thus, based on the above argument, we propose the following hypothesis:

H3. Excessive use of WeChat significantly and positively affects knowledge sharing.

### Knowledge Sharing and Creativity

Knowledge sharing is an important aspect of knowledge management and refers to the behavior of people sharing information and ideas with others (Elrehail et al., 2016). Knowledge sharing is also considered a process in which individuals transmit information across boundaries and aggregate knowledge to create new ideas (Eidizadeh et al., 2017). Extensive use of social media enables knowledge to be widespread in virtual network communities, which promotes knowledge-sharing activities in people's social networking (Kwahk and Park, 2016). Such knowledge-sharing activities trigger creative ideas by arousing divergent thinking (Rhee and Choi, 2017). Thus, using social media for communication promotes individuals' creativity because it stimulates knowledge flow and acquisition from social networks. Knowledge-sharing activities turn tacit knowledge into explicit knowledge (Rhee and Choi, 2017), which helps individuals adopt creative methods to solve problems and innovate best practices from others' experiences (Edwards et al., 2017). Individuals exchange their thoughts, ideas, opinions, and emotions with others through using social media. Individuals benefit by learning from each other. Therefore, we propose the following hypothesis:

H4: Knowledge sharing significantly and positively affects creativity.

### Excessive Use of WeChat and Creativity

Of note, the social function part of WeChat is one representative part. WeChat is not “just” a social media platform but also a platform for acquiring various information. For example, *WeChat-Public-Account* is a channel for users to obtain information when they follow public accounts (Montag et al., 2018). In daily WeChat use, individuals often follow public accounts in which they are interested. Public accounts transmit information regularly to users who follow the account, allowing individuals to understand and store information by continuously reading the content sent from public accounts. Individuals who spend too much time on WeChat will keep reading what they are interested in. Some public accounts update content daily, every few days or weekly. Excessive use of WeChat enables users to follow information provided by different public accounts; thus, they continue learning. Kind and Evans (2015) stated that social media can be considered lifelong learning by enabling people to continue learning over time to stimulate and foster creativity. Therefore, we propose the following hypothesis:

H5: Excessive use of WeChat significantly and positively affects creativity.

**Figure 1:**
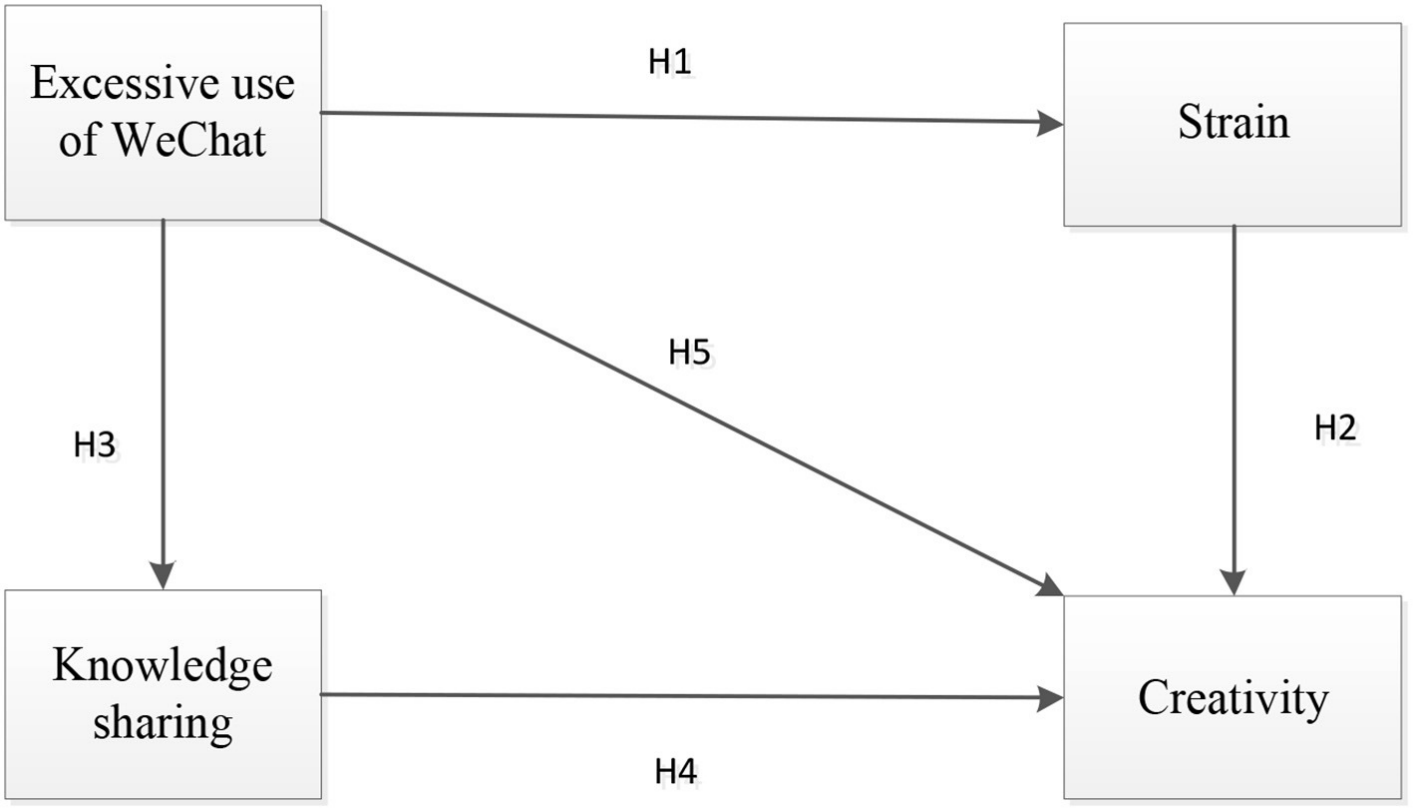
Research model.

Drawing upon the above discussion, Figure 1 shows the research model. It displays the different paths for excessive use of WeChat influencing creativity. Excessive use of WeChat not only has direct influence on creativity but also has indirect impact on creativity through strain and knowledge sharing.

## Methods

### Sample and Procedures

This study used a quantitative research method. An online questionnaire was conducted to test the hypotheses. To improve sample quality and response rates, we employed a professional market research firm to collect data. This firm is a leader in market research firms to provide the service of questionnaire design and data collection. This firm provides required samples from their panel, which has more than 2,600,000 members to participate in various research projects via strict recruiting methods.

This study explored the influence of excessive WeChat use on individual creativity in the workplace. Actually, it aims to explore user behavior, and the target respondents were Chinese employees who use WeChat at work. Before the survey, we made special request for the market research company to choose enterprise staffs as required samples. During the survey, we provided a brief description to explain the research objective and illustrate that all questions mentioned in survey are considering the actual conditions in work context. Then, we sent the questionnaire to enterprise staffs from the required samples provided by the research company. They were told to choose social media they most frequently used in daily work includes WeChat, QQ, DingDing, Weibo, TIM, Zhihu, and others. Only those who self-reported as using WeChat frequently in organizations were valid for this study. The Chinese multinational company Tencent Holding Limited released WeChat in 2011. As an instant messaging software, every registered user obtained the copyright permissions to use WeChat. All respondents are WeChat users in this study; we confirm that the appropriate permissions have been obtained for using WeChat. To control the quality, each respondent submits only one response, and invalid questionnaires are systematically screened and manually rechecked. Finally, we collected 429 completed responses, and 56 invalid responses were eliminated systematically. Besides, we eliminated nine responses from employees who indicated different attitudes for similar items. In fact, we obtained 364 valid responses for the sample. Table 1 shows the demographic details of respondents.

**Table 1:** Respondents' demographics.

**Demographics**	**Items**	**Percent (%)**
Gender	Male	45.6
	Female	54.4
Age (years)	21–25	11.5
	26–30	31.0
	31–35	37.4
	≥35	20.1
Education level	Associate degree or below	13.8
	Bachelor	77.4
	Mater/PhD	8.8
Work experience (years)	≤1	4.1
	1–3	17.6
	3–5	24.2
	5–7	26.4
	≥7	27.7
Industry type	Computers	14.3
	Education	8.2
	Manufacturing	33.0
	Service	14.5
	Banking/finance	9.1
	Construction	4.7
	Health care	6.9
	Others	9.3

### Measures

The tested scales used in this study were adapted from validated scales of previous studies. Suitable modifications were made to fit the new context of the current study. All items were scored using a five-point Likert scale, ranging from 1 “strongly disagree” to 5 “strongly agree.” The measurement items used are presented in the Supplementary Material. Excessive WeChat use was measured using a 4-item WeChat excessive use scale developed by Hou et al. (2017) to assess the degree to which individuals used WeChat. One item was “I feel the need to use WeChat with increasing amounts of time to achieve satisfaction.” A 4-item measurement scale was adapted from Moore (2000) and Ayyagari et al. (2011) for assessing strain. One item was “I feel drained from activities that require me to use social media.” We modified it into “I feel drained from activities that require me to use WeChat” to fit our study context. Knowledge sharing was measured on a 7-item scale adapted from Lu et al. (2006) and Bock and Kim (2002) to assess the extent to which participants shared their knowledge with their companions. One item was “In daily work, I take the initiative to share my work-related knowledge with my colleagues.” Employee creativity was measured with a 3-item scale reported by Durcikova et al. (2011). One item was “I believe I am usually very creative in my solutions to work problems.”

Individual characteristics may affect employee creativity in the workplace. Therefore, this study included some control variables. Individuals' job tenure was considered as a control variable because it can affect creativity (Shin and Zhou, 2007). Demographic diversity (Shin and Zhou, 2007) may also influence creativity; hence, individuals' age, gender, education, and industry type were also viewed as control variables.

### Statistical Analysis

Following the two-step approach of Anderson and Gerbing (1988), this study used the covariance-based structural equation model (CB-SEM) to verify research models and hypotheses. First, construct validity of the measurement model was assessed with confirmatory factor analysis (CFA). Then, the proposed research model was examined by testing SEM. In particular, this study focuses on causal testing, which satisfied the CB-SEM usage criteria. Comparing with partial least square SEM, which focuses on causal prediction, CB-SEM is applicable to causal path analysis. Therefore, the CB-SEM was used as an analysis tool and Mplus 7.4 was adopted.

Several statistical methods were used to test the potential common method bias, which exists in self-reported data (Podsakoff et al., 2003). First, an exploratory principal axis factoring analysis without rotation was applied to all multi-item measures (Malhotra et al., 2006) in IBM SPSS 21.0. If one single factor accounted for most of the covariance in the independent and dependent variables, the common method bias existed (Podsakoff et al., 2003). However, in current research, one factor explained only 28% of the variance, indicating that the potential common method bias was unlikely to occur in this study. Second, this study assessed the correlation matrix as per the procedure suggested by Pavlou et al. (2007). The correlation matrix (Table 2) indicated that no correlation between the variables exceeded the threshold of 0.90 (Bagozzi et al., 1991). Therefore, no common method bias occurred in the data of this study. We also conducted collinearity diagnostics to detect multicollinearity in the research model. The variance inflation factors for all constructs were near 1, which is less than the threshold of 5 (Hair et al., 2011). Thus, no collinearity occurred among any constructs.

**Table 2:** Correlation matrix among study variables.

	**1**	**2**	**3**	**4**	**5**	**6**	**7**	**8**	**9**
Gender									
Age	−0.152^**^								
Education level	−0.034	−0.045							
Working experience	−0.210^**^	0.643^**^	−0.119^*^						
Industry type	0.063	0.083	0.014	−0.077					
EWUS	0.106^*^	0.056	−0.008	0.087	−0.046				
Strain	−0.024	−0.085	0.126^*^	−0.158^**^	−0.012	−0.056			
Knowledge sharing	0.021	0.081	0.004	0.121^*^	−0.062	0.113^*^	−0.264^**^		
Creativity	−0.116^*^	−0.006	0.080	0.076	−0.074	0.217^**^	−0.090	0.382^**^	

* *p < 0.05*,

** *p < 0.01*.

*EWUS, excessive use of WeChat*.

## Results

### Measurement Model

Different forms of instrument validity and reliability were accessed in this study. Based on the criteria suggested by Fornell and Larcker (1981), construct reliability was accessed by determining whether Cronbach α and composite reliability (CR) are >0.7. Convergent validity was evaluated by the item loadings of the questionnaire on the respective constructs and the average variance extracted (AVE). Many researchers suggest that item loading is not <0.6 (Fornell and Larcker, 1981; Van Van Dyne et al., 2002). Meanwhile, the AVE is >0.50 (Fornell and Larcker, 1981). Table 3 shows the Cronbach α and CR for all constructs are >0.70. The AVE of each construct is larger than 0.50, and all item loadings ranged from 0.61 to 0.85. Thus, construct reliability and convergent validity of our measurement instrument were acceptable because all measures met the recommended levels. Discriminant validity was assessed by checking if the square root of AVE for each construct was larger than the correlations between the construct and all other constructs (Fornell and Larcker, 1981). Table 4 shows construct correlation matrix and the square root of AVE, which indicates that the square root of AVE exceeds correlations with other constructs. It demonstrated confirmation of sufficient discriminant validity for study measures. Construct validity can be assessed by a fit index through CFA. All reflective latent variables were tested via CFA, and the fit statistics for this model were subjected to common cutoff criteria. Table 5 shows the results of the CFA. The χ^2^/degree of freedom ratio was 1.79, which is <5. The comparative fit index (CFI) and Tucker–Lewis index (TLI) scores were >0.90, and the standardized root mean square residual (SRMR) was <0.08. The root mean square error of approximation (RMSEA) was <0.1. The Akaike information criterion and Bayesian information criterion scores were small and met the criteria of a good model of fit, indicating that the CFA model fit well.

**Table 3:** Mean, SD, reliability, and convergent validity.

**Construct**	**Item**	**Mean**	**SD**	**Standard loading**	**Cronbach α**	**CR**	**AVE**
Excessive use of WeChat (EWUS)	EWUS1	3.52	1.094	0.70	0.801	0.80	0.5100
	EWUS2	3.21	1.150	0.61			
	EWUS3	3.17	1.105	0.85			
	EWUS4	3.19	1.226	0.67			
Strain	Strain1	2.77	0.989	0.66	0.845	0.85	0.5844
	Strain2	2.54	1.016	0.81			
	Strain3	2.71	1.178	0.73			
	Strain4	2.55	1.068	0.84			
Knowledge sharing (KS)	KS1	3.73	0.717	0.78	0.887	0.89	0.5310
	KS2	3.93	0.834	0.68			
	KS3	3.98	0.782	0.73			
	KS4	3.79	0.870	0.77			
	KS5	3.78	0.901	0.72			
	KS6	3.90	0.754	0.74			
	KS7	3.76	0.843	0.68			
Creativity	Creativity1	3.81	0.863	0.70	0.775	0.78	0.5358
	Creativity2	3.75	0.856	0.76			
	Creativity3	3.60	0.829	0.74			

**Table 4:** Construct correlation matrix and the square root of AVE in the diagonal.

**Construct**	**EWUS**	**Strain**	**KS**	**Creativity**
EWUS	0.714			
Strain	−0.056	0.764		
KS	0.113	−0.264	0.729	
Creativity	0.217	−0.090	0.382	0.732

*EWUS, excessive use of WeChat; KS, knowledge sharing*.

**Table 5:** Fit indices for the estimated model.

	**χ^**2**^**	***df***	**χ^**2**^/ *df***	**P**	**AIC**	**BIC**	**RMSEA**	**CFI**	**TLI**	**SRMR**
CFA	230.8	129	1.79	0.00	15237.9	15471.8	0.047	0.96	0.95	0.045
Research model	517.6	134	3.86	0.00	15514.8	15729.2	0.089	0.86	0.84	0.316

### Structural Model

**Figure 2:**
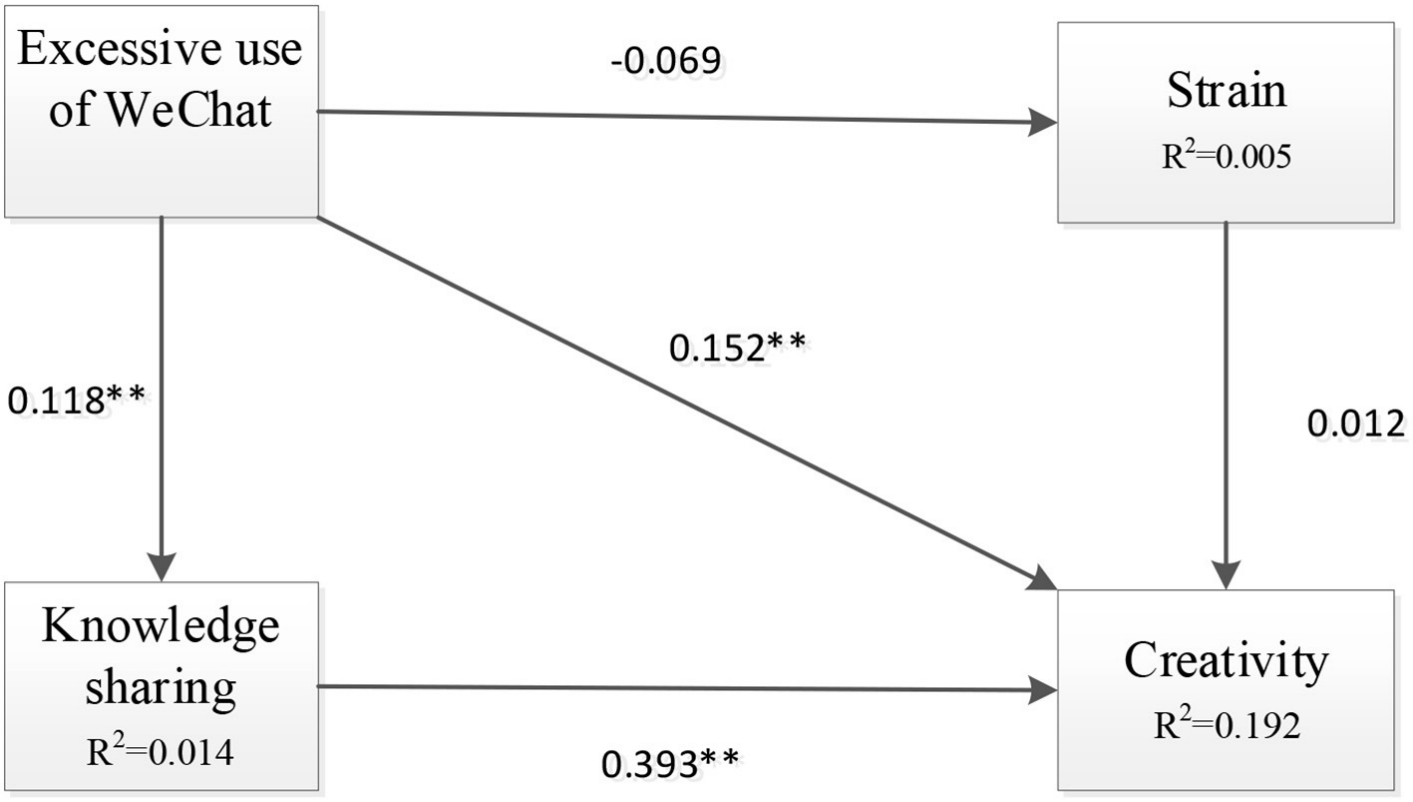
Results of the structural model. *p < 0.05; **p < 0.01.

The structural model results are illustrated in Figure 2. The proposed hypotheses in this research were tested via bootstrap resampling estimation, which is the best method for testing mediating effects (Preacher and Hayes, 2008). Compared with other methods to test meditating effects, bootstrap resampling estimation has higher statistical validity (Cheung and Lau, 2008; Williams and MacKinnon, 2008). Table 5 shows the fit statistics were acceptable according to the recommended criteria (χ^2^/*df* <5, RMSEA <0.1, CFI >0.8, TLI >0.8, SRMR <0.5). Steiger (1990) believed that RMSEA <0.1 is a good fit, whereas <0.05 indicates a very good fit. RMSEA value >0.1 indicates poor fit (Browne and Cudeck, 1992), and range from 0.08 to 0.1 indicates mediocre fit (MacCallum et al., 1996). The values of CFI and TLI range from 0 to 1; the values closer to 1 are greater (Hu and Bentler, 1999). The fit statistics were subjected to the criteria of a good model of fit.

If the fit of the model is adequate, scholars suggested that testing model should combine the consideration of research purpose, theoretical background (Barrett, 2007; Bollen, 2011), and the significance of parameter estimates (Cole and Maxwell, 2003), such as the factor loadings for effect indicators and the regression coefficients for causal indicator (Bollen, 2011) and the confidence intervals around key parameter estimates (Cole and Maxwell, 2003). Therefore, the structural model results were assessed via the estimated path coefficients with asterisks. The results showed that H3, H4, and H5 were supported, but H1 and H2 were not. Specifically, excessive use of WeChat significantly and positively affected creativity [β = 0.152, BC95% (0.065–0.234); *p* = 0.001] and knowledge sharing [β = 0.118, BC95% (0.029–0.203); *p* = 0.009]. However, its effect on strain was not significant [β = −0.069, BC95% (−0.186–0.040); *p* = 0.239]; thus, H1 was rejected. Knowledge sharing was significantly and positively correlated with creativity [β = 0.393, BC95% (0.272–0.498); *p* = 0.000], but the impact of strain on creativity was not significant [β = 0.012, BC95% (−0.078–0.098); *p* = 0.800]. Figure 2 presents the structural model with standardized path coefficients. Table 6 provides the bootstrapping estimates for path analysis, including the confidence intervals.

**Table 6:** Standardized path coefficients, standard deviation, and 95% CI.

**Hypothesis**	**Path**	**Standardized path coefficients**	***t*-value**	***p*-value**	**SD**	**95% CI**	**Include 0**	**Result**
H1	EWUS → strain	−0.069	−1.179	0.239	0.059	[−0.186,0.040]	Yes	Not support
H2	Strain → creativity	0.012	0.254	0.800	0.046	[−0.078,0.098]	Yes	Not support
H3	EWUS → KS	0.118	2.614	0.009	0.045	[0.029,0.203]	No	Support
H4	KS → creativity	0.393	7.002	0.000	0.056	[0.272,0.498]	No	Support
H5	EWUS → creativity	0.152	3.441	0.001	0.044	[0.065,0.234]	No	Support

*EWUS, excessive use of WeChat; KS, knowledge sharing*.

In general, the structural model results indicated effective hypothesis testing. Based on the person–environment fit model and motivation theory, this study examined the influence of excessive WeChat use at work on individuals' creativity. The results indicated that excessive use of WeChat significantly and positively affects employee creativity. Meanwhile, excessive use of WeChat also positively affects creativity through knowledge sharing. Hence, some hypotheses are verified in this study.

## Discussion and Implications

### Discussion

This study attempts to investigate the underlying mechanism of the different paths by which excessive use of WeChat influences individual creativity. Drawing on discoveries in social media research, this study empirically examined the direct and indirect impact of excessive use of WeChat on individual creativity. The indirect impact focuses on how excessive use of WeChat promotes or restrains individual creativity through knowledge sharing and psychological strain. Several key findings can be derived from the results of this study.

First, the relationship between excessive use of WeChat and strain is not significant, whereas excessive use of WeChat has significantly positive influence on knowledge sharing. The direction of these relations is consistent with our prediction of H3 but opposite of H1. It indicates that excessive use of WeChat enables individuals spending a lot time and energy to share knowledge, which is consistent with previous study results indicating that social media enables individual knowledge sharing (Marianna and Kalotina, 2015). Meanwhile, this result also accords with the function of social media, which as a tool of social interaction allowing individuals to create, share, and exchange ideas and knowledge (Zeng and Gerritsen, 2014). The results showed excessive use of WeChat did not have significant relationship with psychological strain. This result is inconsistent with evidence from previous research, which found that excessive use of social media at work leads to social media exhaustion (Yu et al., 2018). This result may be explained from two points. The first point is that employees use WeChat mainly related to personal purposes such as entertainment and socialization. They use WeChat for gaming to take a break and for socialization to develop work relationships. Such personal activities can bring instant gratification and pleasure (Cao and Yu, 2019). Furthermore, individuals' use of social media to socialize is a benefit for their personal relationship development (Shiau et al., 2017) and information sharing with others (Schmidt et al., 2016), thereby obtaining work support to a certain extent (Cao and Yu, 2019). The second point is that employees may view excessive use of WeChat as a “harmless” habit that can meet their superficial needs (Cao and Yu, 2019). By this way, excessive use of WeChat is a habitual activity, which pursues gratification and enjoyable experiences (Wang et al., 2015). These situations may be the reason why the influence of excessive use of WeChat on strain is not significant.

Second, the result does not support H2 but prove H4. Compared to the result that strain has no negative impact on employee creativity, knowledge sharing significantly and positively affects employee creativity. For the former, one plausible reason is the role of strain. Van Dyne et al. (2002) have explored the influence of work strain and home strain on employee sales performance and employee creativity. They found that work strain was not related to employee creativity, whereas home strain had a significant and negative impact on employee creativity (Van Dyne et al., 2002). Work strain refers to conflict or tension occurs in workplace with colleagues and supervisors (Van Dyne et al., 2002). In this study, the survey focuses on the actual conditions in work context. Hence, the result indicating there is no significant relationship can be explained. For the latter, knowledge sharing as a key factor influencing creativity is consistent with most previous studies, which indicate that knowledge sharing significantly predicts employee creativity (Marianna and Kalotina, 2015; Eidizadeh et al., 2017; Liao and Chen, 2018). Our study thereby enriches the related research.

Finally, the result indicated a positive outcome of excessive use of WeChat. Excessive use of WeChat significantly and positively predicted employee creativity, which supports H5. It suggested that spending excessive time and energy on WeChat can improve employee creativity. In previous study, a lot of studies explored the dark side of excessive social media use. Excessive use of social media contributes to addictive behaviors (Lian et al., 2019); job performance reduced (Yu et al., 2018; Cao and Yu, 2019). Few studies attempted to explore an upside to excessive use of social media. According to the argument of Kind and Evans (2015), they demonstrated that social media can be viewed as a tool of lifelong learning. Meanwhile, knowledge sharing on social media in daily life is an actual situation. So, we try to examine the positive influence of excessive use of social media. Our study thereby provides additional evidence of the social media usage. We make the case that this finding significantly contributes to the bright side of excessive-use behavior by theoretically and empirically investigating the positive outcomes of excessive use of WeChat on creativity.

### Theoretical Implications

This research provides several important theoretical implications. First, unlike prior studies that focused on the dark side of excessive social media use in the organizational environment (Yu et al., 2018; Cao and Yu, 2019; Lian et al., 2019), this study contributes to the extant social media research by exploring the potential positive consequences of excessive social media use. Compared with previous research on excessive use of social media and the most popular social media platforms in Western countries, this study researched the excessive use of WeChat in Chinese context. Although previous studies found that excessive-use behaviors on social media can induce negative outcomes such as decreased job performance (Yu et al., 2018). However, whether excessive-use behavior always leads to bad outcome should be systematically investigated. This study emphasized the positive impact of excessive social media use in the workplace to obtain balance perspectives of excessive social media usage.

Second, the present research extends the theoretical understanding of the P-E fit model by theoretically examining the relationship between excessive use of social media and psychological strain. However, excessive use of WeChat was found no significantly relationship with strain of social media usage. The result departs from the P-E fit model, which is inconsistent with other studies that indicated social media overuse related to strain of mobile technology (Zheng and Lee, 2016). In essence, the P-E fit model explained the misfit between the person and the environment relying on individual subjective evaluation (Edwards and Van Harrison, 1993). The extent of the excessive use of WeChat for personal or work-oriented purposes does not break the equilibrium state based on individual evaluation. It indicated that the demand of WeChat use matches the user's energy and time to use.

Third, this research highlights the underlying mechanism of the different paths by which excessive use of WeChat influences employee creativity. Based on the P-E fit model and transactional theory of stress and coping, this study developed a theoretical model and empirically examined the three paths by which excessive use of WeChat influences employee creativity. Excessive use of WeChat positively influences employee creativity through the effect on knowledge sharing. Meanwhile, excessive use of WeChat directly influences employee creativity. This finding further advances our theoretical understanding of the relationship between excessive WeChat use and employee creativity. Particularly, even if excessive use of social media will decrease individuals job performance (Cao and Yu, 2019), individuals being excessively engaged in different social media may lead to different outcomes.

### Practical Implications

This research also provides empirical evidence that excessive use of WeChat in the workplace may positively influence employee creativity through different paths. Thus, employees and organizations should not ignore the seemingly common excessive use of WeChat. This research offers several practical implications as follows.

First, employees who spend a lot of time using WeChat should be wise to its positive consequences. They should realize that they can communicate with others to obtain knowledge. For example, employees can ask coworkers or friends questions in their social network and request them to share their knowledge. Furthermore, if employees are in marketing or sales positions where they often use WeChat to keep customer, they can utilize WeChat to collect customers' feedback. For instance, employee can know the interest of potential customer through frequent communication and interaction. Besides, employee should be aware that using WeChat to follow own interests is associated with improved creativity. For example, employees follow some own-interest public accounts. They can get hold of large amounts of fragmented knowledge through constantly focusing.

Second, in accordance with previous research, this study showed that excessive use of WeChat promotes creativity directly and improves employee creativity indirectly through knowledge sharing. Organizations should realize that social media provides a platform for individuals to acquire information and knowledge (Panahi et al., 2016a; Alshahrani and Pennington, 2018). Organizations can encourage employees to communicate on WeChat instead of not using. Organizations also should be aware that excessive use of WeChat may have positive consequences for employees. In the current work environment, organizations cannot simply separate employees' social and work-related use of WeChat, but organizations can also formulate policies to regulate the use pattern in workplace and thereby enhance employee creativity. Organizations can support employees by joining the online community of practice based on social media technology. Community of practice is a group that interacts regularly to share knowledge and experiences (Amin and Roberts, 2008). It motivates individuals to participate in collective knowledge generation by providing an opportunity for continuous and timesaving learning (Bandow and Gerweck, 2015). By setting up a community of practice that is based on WeChat group chatting for employees who work in similar jobs, members can share tips and tools, ask questions, and come up with answers online, which may help improve employees' creative performances.

### Limitations and Future Research

This study had several limitations. First, this study used excessive use of WeChat social media as the omnibus measure for research. Future studies can explore the different dimensions for excessive use of WeChat based on social media use patterns, such as cognitive use, hedonic use, and social use (Ali-Hassan et al., 2015). Clearly distinguishing between work and private use can enhance our understanding of excessive WeChat use. Second, this study advanced the understanding of the relationship between excessive WeChat use and employee creativity. However, individual differences in WeChat use may be associated with personality traits such as the “big five” personality traits (Montag et al., 2018). Future research may focus on the importance of individual differences in excessive-use behavior and individual creativity or other performance factors. Third, the data collected for our research were from Chinese participants. The Chinese culture of high collectivism, high-power distance (Geert-hofstede, 2017), and Guanxi (relationship) culture (Chen et al., 2017), which is a unique characteristic of the Chinese society, may exert an extra effect on individual social interactions. Hence, users may be willing to share information, provide social support, and maintain communication on WeChat. Because of cultural differences, future research should test the model on different cultures to increase the generalizability of these findings. Finally, this research relied on self-reporting techniques, which can easily generate bias. Future research should consider more objective and quantitative assessment methods to collect exact information on what individuals do on WeChat. For example, the methods of psychoinformatics could be used to obtain exact information on individual usage behaviors on WeChat (Montag et al., 2018).

## Conclusion

This research explored the phenomenon of excessive WeChat use and investigated the different paths by which it influences creativity in the work environment. To empirically assess the research model, a survey was conducted of 364 Chinese WeChat users working for organizations. Some of the proposed hypotheses were supported. The results demonstrated that excessive WeChat use can positively affect creativity, which is indirectly influenced through knowledge sharing. Meanwhile, excessive use of WeChat directly influences employee creativity. These research findings help enrich social media research and improve the current understanding of how excessive social media use influences individuals and the implications of its influence.

## Data Availability Statement

The original contributions presented in the study are included in the article/Supplementary Material, further inquiries can be directed to the corresponding author.

## Ethics Statement

Ethical review and approval was not required for the study on human participants in accordance with the local legislation and institutional requirements. Written informed consent for participation was not required for this study in accordance with the national legislation and the institutional requirements. Written informed consent was implied via completion of the survey.

## Author Contributions

HQZ performed the research design and outline of manuscript. MW contributed to drafting the manuscript and analyzing questionnaire data. ML collected the data and improved the empirical analysis. XDC revised the draft. All authors contributed to the article and approved the submitted version.

## Conflict of Interest

The authors declare that the research was conducted in the absence of any commercial or financial relationships that could be construed as a potential conflict of interest.
