# Supplementary material for: Excessive Use of WeChat at Work Promotes Creativity: The Role of Knowledge Sharing, Psychological Strain
Source: Front Psychol. 2021 Mar 18;12:571338. doi: 10.3389/fpsyg.2021.571338 (PMC8012808; doi:10.3389/fpsyg.2021.571338)
Supplement: Supplementary file 1 [file Table_1.pdf]

Appendix

Appendix: Measurement items adopted

Constructs	Items	Source
Excessive use of WeChat	I find myself saying "just a few more minutes" when browsing WeChat	(Hou, et al., 2017)
	There are times when I would rather play on WeChat than go out with my friends	
	I feel the need to use WeChat with increasing amounts of time to achieve satisfaction	
	I have slept late because of preoccupation with WeChat	
Strain	I feel drained from activities that require me to use WeChat	Moore(2000);
	I feel tired from my social activities on WeChat	Ayyagari et al. (2011)
	Working all day with WeChat is a strain for me	
	I feel burned out from my social activities on WeChat	
Knowledge sharing	In daily work, I take the initiative to share my work-related knowledge to my colleagues	Lu et al. (2006);
	I keep my work experience and never share it out with others easily (R)	Bock & Kim (2002)
	I share with others useful work experience and know-how	
	After learning new knowledge useful to work, I promote it to let more people learn it	
	I never tell others my work expertise unless it is required in the company(R)	
	In workplace I take out my knowledge to share with more people	
	I actively use IT sources available in the company to share my knowledge	
Creativity	Innovativeness is a must in my work	(Durcikova, et al., 2011)
	Most of the time I am quite innovative in solving work problems	
	I believe I am usually very creative in my solutions to work problems	

(R): Reverse coded item
